# Novel *CYP4F22* mutations associated with autosomal recessive congenital ichthyosis (ARCI). Study of the *CYP4F22* c.1303C>T founder mutation

**DOI:** 10.1371/journal.pone.0229025

**Published:** 2020-02-18

**Authors:** Uxia Esperón-Moldes, Manuel Ginarte-Val, Laura Rodríguez-Pazos, Laura Fachal, Ana Martín-Santiago, Asunción Vicente, David Jiménez-Gallo, Encarna Guillén-Navarro, Loreto Martorell Sampol, María Antonia González-Enseñat, Ana Vega

**Affiliations:** 1 Fundación Pública Galega de Medicina Xenómica-SERGAS, Grupo de Medicina Xenómica-USC, CIBERER, IDIS, Santiago de Compostela, Spain; 2 Departamento de Ciencias Forenses, Anatomía Patolóxica, Xinecoloxía, Obstetricia e Pediatría, Universidade de Santiago de Compostela, Santiago de Compostela, Spain; 3 Dermatology Service of Complexo Hospitalario Universitario de Santiago, Santiago de Compostela, Spain; 4 Dermatology Service of Complexo Hospitalario Universitario de Vigo, Vigo, Spain; 5 Dermatology Service of Hospital Universitari Son Espases, Palma de Mallorca, Spain; 6 Dermatology Service of Hospital Sant Joan de Déu, Barcelona, Spain; 7 Dermatology Service of Hospital Puerta del Mar, Cádiz, Spain; 8 Dermatology Service of Hospital clínico universitario Virgen de la Arrixaca, Murcia, Spain; 9 Genetic Service of Hospital Sant Joan de Déu, Barcelona, Spain; Central South University Third Xiangya Hospital, CHINA

## Abstract

Mutations in *CYP4F22* cause autosomal recessive congenital ichthyosis (ARCI). However, less than 10% of all ARCI patients carry a mutation in *CYP4F22*. In order to identify the molecular basis of ARCI among our patients (a cohort of ninety-two Spanish individuals) we performed a mutational analysis using direct Sanger sequencing in combination with a multigene targeted NGS panel. From these, eight ARCI families (three of them with Moroccan origin) were found to carry five different *CYP4F22* mutations, of which two were novel. Computational analysis showed that the mutations found were present in highly conserved residues of the protein and may affect its structure and function. Seven of the eight families were carriers of a highly recurrent *CYP4F22* variant, c.1303C>T; p.(His435Tyr). A 12Mb haplotype was reconstructed in all c.1303C>T carriers by genotyping ten microsatellite markers flanking the *CYP4F22* gene. A prevalent 2.52Mb haplotype was observed among Spanish carrier patients suggesting a recent common ancestor. A smaller core haplotype of 1.2Mb was shared by Spanish and Moroccan families. Different approaches were applied to estimate the time to the most recent common ancestor (TMRCA) of carrier patients with Spanish origin. The age of the mutation was calculated by using DMLE and BDMC2. The algorithms estimated that the c.1303C>T variant arose approximately 2925 to 4925 years ago, while Spanish carrier families derived from a common ancestor who lived in the XIII century. The present study reports five *CYP4F22* mutations, two of them novel, increasing the number of *CYP4F22* mutations currently listed. Additionally, our results suggest that the recurrent c.1303C>T change has a founder effect in Spanish population and c.1303C>T carrier families originated from a single ancestor with probable African ancestry.

## Introduction

Autosomal recessive congenital ichthyosis (ARCI) is a group of non-syndromic rare diseases that affect keratinization. They can be divided into three main clinical subtypes: lamellar ichthyosis (LI; OMIM 242300), congenital ichthyosiform erythroderma (CIE; OMIM 242100) and harlequin ichthyosis (HI; OMIM 242500). Minor variants include bathing suit ichthyosis (BSI) and two types of self-improving ichthyosis, self-healing collodion baby (SHCB) and acral self-healing collodion baby (ASHCB). ARCI is characterized by epidermal scaling over the whole body which is usually accompanied by other symptoms such as presence of collodion membrane at birth, ectropion, eclabium, alopecia, palmar-plantar hyperkeratosis, hypohidrosis and variable erythema, among others [1,2]. To date, mutations in eleven different genes have been found to underlie ARCI: *ABCA12*, *ALOX12B*, *ALOXE3*, *CYP4F22*, *NIPAL4*, *TGM1* and more recently *CERS3*, *PNPLA1*, *CASP14*, *SDR9C7* and *SULT2B1* [3–8]. These genes encode proteins that participate in the proper functioning of the skin barrier through various pathways involved in lipid metabolism, its transport within the stratum corneum, and the formation of the cornified envelope by cross-linking and lipid attachment [9]. *TGM1* mutations are the main cause of ARCI, followed by *ALOXE3* and *ALOX12B*. Mutations in *ABCA12*, *NIPAL4* and *CYP4F22* are less frequent, while the rest of genes are much rarer causes of ARCI. However, there is still a percentage of ARCI cases without an identifiable cause (15% of cases approximately)[10]. A potential genotype-phenotype correlation has not been found in any gene with the exception of *TGM1* and *ABCA12*. Mutations in *TGM1* have been demonstrated to be significantly associated with the presence of collodion membrane at birth, ectropion, plate-like scales, and alopecia [11]. *ABCA12* is the only gene associated with the harlequin ichthyosis phenotype. It has been suggested that the type of mutation is correlated with the ARCI phenotype: homozygotes or compound heterozygotes with truncating *ABCA12* mutations lead to the harlequin ichthyosis phenotype whereas missense mutations result in the LI and CIE phenotypes [12].

CYP4F22 (OMIM 611495) is a protein that belongs to heme-thiolate cytochrome P450 subfamily 4 (CYP4), and is predominantly active in lipid metabolism. The gene that encodes this protein spans 44 kb and is comprised of 14 exons and 531 amino acids. Its transcript is present in various tissues but is highly expressed in the epidermis, including skin and keratinocytes [13]. A recent study demonstrated that CYP4F22 is a ω-hydroxylase which is essential for acylceramide synthesis, which in turn is important for epidermal barrier formation [14]. Consistent with this, mutations in *CYP4F22* have been identified in LI, CIE and SHCB ARCI patients [15,16].

Despite its known implication in the development of ARCI, *CYP4F22* is one of the least frequently reported ARCI genes along with *CERS3*, *NIPAL4* and the very recently described *SULT2B1*, *SDR9C7* and *CASP14*. In order to identify the molecular basis of ARCI among our patients, we performed a mutational screening through direct Sanger sequencing in combination with a multigene NGS panel in a cohort of ninety-two Spanish ARCI individuals. In this study, we report the mutations found in *CYP4F22*. Interestingly, one of the identified mutations, c.1303C>T; p.(His435Tyr), was present in most of the families with mutated *CYP4F22*, suggesting a possible founder effect.

Few founder mutations have already been described in the Spanish population. Our group demonstrated the existence of founder effects in two different genes, *PNPLA1* and *ABCA12* [17,18]. In Galicia, three founder mutations, c.2278C>T, c.1223_1227delACAC and c.984+1G>A, account for the majority of all *TGM1* mutations identified in this Northwestern Spanish region [19]. Interestingly, the same founder mutation identified in Spain, *ABCA12*: c.4139A>G, also has founder effects in other populations, including Morocco and Algeria [20]. However, no other founder mutations have been reported in the Moroccan ARCI population.

The purpose of the study is: I) to identify the underlying genetic mutations in ARCI families accompanied by their clinical characteristics in an attempt to find a possible genotype*-*phenotype association, II) to study the potential effect of those mutations on protein structure and function, III) to elucidate whether the high frequency of the c.1303C>T variant is due to a founder effect and, IV) to estimate the time to the most recent common ancestor (TMRCA) of all carrier families and the origin of this mutation.

## Materials and methods

### Patient recruitment and clinical characterization

Ninety-two Spanish ARCI patients, who belonged to eighty-two different families, 49 males, 42 females and one patient with no sex information, participated in the study. All patients were Spanish, but nine patients from eight families had non-Spanish ancestries. Six patients had ancestries from Morocco, one from Poland, one from Cuba and one from Venezuela. The average age of patients at the time of the study was 27 years old. One hundred and fifty-nine healthy close relatives were also included.

Affected individuals were clinically characterized by a dermatologist. Pedigrees of families with at least three generations were also required where possible. Available pedigrees of families with mutations in *CYP4F22* are presented as supplementary material S1 Fig.

Informed consent was obtained from each subject. The study was approved by the Galician Ethical Committee for Clinical Research (Code 2013/056) and the procedures followed were in accordance with the Declaration of Helsinki. All participants provided written informed consent.

### Mutation screening

Genomic DNA was extracted from peripheral blood from each patient and available close relatives according to standard procedures. Mutation analysis of *CYP4F22* was performed by Sanger sequencing or targeted resequencing on SOLiD 5500xl or Ion Proton Platforms (Thermo Fisher Scientific; San Jose, CA, USA) according to manufacturer’s protocols. Sequencing library preparation was performed according to Agilent SureSelect (Agilent Technologies, Santa Clara, CA, USA) protocols. Variants were validated by Sanger sequencing and annotated according to NCBI Reference Sequence: NM_173483.3.

### Computational analyses

*In silico* analyses were performed using Alamut Visual Interactive Biosoftware 2.11.0 (Alamut, Rouen, France), which predicts the possible functional impact of mutations. Within Alamut, the pathogenicity of novel missense variants was tested by AlignGVGD, SIFT and Mutation Taster, while HSF, MaxEnt and NNSPLICE were used to study the possible splicing effect of the splice-site variants. In addition, we used the aggregate scorer for variants of unknown significance, CADD (Combined Annotation Dependent Depletion; *https://cadd.gs.washington.edu*).

The degree of conservation of each residue was assessed using the BLASTP tool available through the Uniprot website (*http://www.uniprot.org/blast*). A multiple sequence alignment was performed in order to compare the human CYP4F22 protein (UniProt: Q6NT55) with seven different species (Gorilla, Cow, Pig, Horse, Mouse, Frog and Fish).

The significant differences in haplotype frequencies in cases and controls were evaluated with the R statistical software version 3.3.2 (R Foundation for Statistical Computing, Vienna, Austria) using contingency table analysis with the Fisher Exact Test.

The secondary mRNA structures and thermodynamic parameters of wild type and mutant *CYP4F22* were predicted using ‘the mfold web server’ using default parameters (*http://mfold.rna.albany.edu/?q=mfold*).

Modeling of CYP4F22 pathogenic variants was performed using the template created by Kumar [21] based on the comparative modeling method. The CYP4F22 model was used to compare the mutant protein with the wild type. We used the Swiss-Pdb Viewer (SPDBV) software (*http://spdbv.vital-it.ch/*) for the study and visualization of the predicted structures.

### Haplotype reconstruction

**Fig 1:**
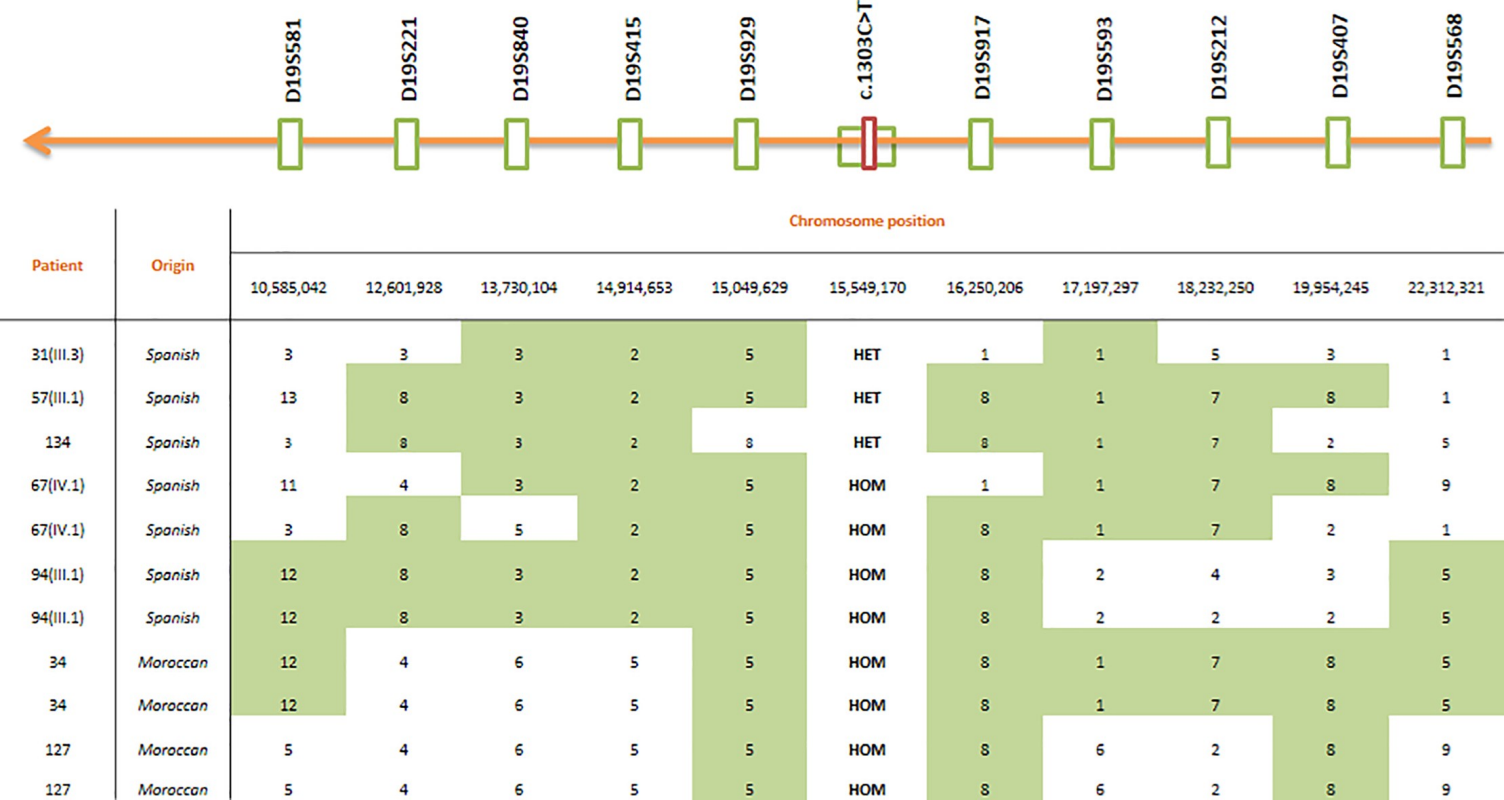
Schematic representation of the ten extragenic markers, their chromosomal position and haplotypes reconstructed for each proband with the c.1303C>T mutation. The founder haplotype identified is shaded light grey. Some patients are homozygous carriers (two haplotypes represented) and some are heterozygous carriers (one haplotype represented).

To test for a possible founder effect, we constructed the haplotypes of individuals harboring the c.1303C>T mutation by using ten extragenic microsatellite markers (Fig 1). Control allele frequencies in the same markers were determined using two hundred chromosomes of Spanish individuals who did not present any type of ichthyosis or other types of epidermal disease. The control group was composed of fifty men and fifty women with an average age of 42 years old.

These markers were selected from the UCSC Genome Browser (*https://genome.ucsc.edu*). They spanned a 12Mb region flanking the *CYP4F22* gene locus, with five microsatellites each side of the gene. Forward PCR primers were labeled with either FAM or HEX fluorescent dyes (Sigma- Genosys Ltd. Cambridgeshire, UK). The oligonucleotide sequences used for genotyping are available in S1 Table. The amplification products were separated by capillary electrophoresis on an ABI3730xL sequencer and analyzed with GeneMapper v4 Software.

To reconstruct the chromosome haplotypes of the 200 controls we used the PHASE v2.11 software (*http://www.stat.washington.edu/stephens/phase/download.html*). Patient´s haplotypes were manually reconstructed by inspection of all marker alleles around the disease locus. The genetic distances between the markers, indicated in Fig 1, were derived from the UCSC database (*https://genome.ucsc.edu/*) (Reference genome GRCh37/hg19). Physical distances were converted to centiMorgans assuming 2cM = 1 Mb according to the deCODE genetic map, also available through the UCSC website.

### Estimation of TMRCA

The TMRCA was estimated based on the linkage disequilibrium, using single marker algorithms and haplotype sharing methods:

-We used three single marker algorithms that produce TMRCA estimates based on the degree of recombination between one marker and the mutation across generations. The algorithms described by Bergman *et al* [22], Risch *et al* [23] and Yan *et al* [24] (applying the correction proposed by Labuda [25] for two different growth rates: Spanish growth rate *r* = 0.0748 and European growth rate *r* = 0.05). To translate map distances into recombination fractions we used the Haldane map function [26]. The single marker estimation for each algorithm was summarized by the mean of the results across the available markers. The D19S415 allele was present in all Spanish origin chromosomes carrying the mutation, thus this marker was not included in the estimations (see Supplementary Information S2 Table for further details).

-We also used two methods based on haplotype sharing. One developed by Gandolfo *et al* [27] based on the genetic length of ancestral haplotypes shared between individuals who carry the mutation; and another created by Genin *et al* [28], which uses both the overall genetic length of the haplotype and the physical distances between markers.

### Mutation age estimation

To estimate the c.1303C>T; p.(His435Tyr) mutation age, two mathematical approaches were applied [29,30]:

- The program BDMC v2.1 (*http://www.rannala.org/docs/bdmcdoc.html*), utilizes a Markov chain method that estimates the age of a given allele though a combination of its frequency and the extent of variation among different copies. Confidence intervals of the MLE (Maximum Likelihood Estimate) were calculated using the asymptotic approach of the maximum likelihood method [31].
- The software DMLE+ v2.3 (*http://dmle.org/*) instead employs a Bayesian estimation of the position of a given disease mutation relative to a set of markers.

For both approaches, the proportion of mutation-bearing chromosomes (*f*) and the population growth parameter (*r*) were required. Considering that the estimate of mutation age seems to be sensitive to demographic parameters (growth rate, mutation frequency and population size), we analyzed our haplotype data assuming the two different growth rates mentioned previously (*r* = 0.0748 and *r* = 0.05) and three different proportions of mutation-bearing chromosomes, according to data published by Hernández-Martín *et al* [32] (*f* = 2.99x10^-4^, *f* = 2.03x10^-4^, *f* = 2.32x10^-4^).

## Results

### *CYP4F22* identified mutations

Eight patients from eight different families, seven new and one previously reported by Noguera-Morel *et al* [15] (Family 67), were carriers of five different *CYP4F22* mutations. Although all probands were born in Spain, three of them were of Moroccan origin (Table 1). Of the six *CYP4F22* mutations identified, four of them were missense mutations and one was a splice-site mutation. Two of these were novel, c.368-1G>A and c.1543C>T, p.(Arg515Cys); and three were previously described by Lefevre *et al* [13] [c.1303C>T; p.(His435Tyr) and c.728G>A; p.(Arg243His)] and Lima Cunha *et al* [33] [c.982G>A; p.(Glu328Lys)]. The novel mutation, c.368-1G>A, could not be confirmed by segregation analysis. The recurrent mutation c.1303C>T; p.(His435Tyr) accounted for 69% of the mutant alleles (eleven of sixteen). Four patients were homozygous carriers while three patients had the mutation in a compound heterozygous form with c.1543C>T, c.728G>A and c.982G>A.

**Table 1:** Clinical and genetic information of patients with *CYP4F22* mutations.

**Patient**^a^		**31(III.3)**	**34**	**57(III.1)**	**67(IV.1)**	**94(III.1)**	**123**	**127**	**134**
**Mutations**	Mutation 1	c.1303C>T p.(His435Tyr)	c.1303C>T p.(His435Tyr)	c.728G>A p.(Arg243His)	c.1303C>T p.(His435Tyr)	c.1303C>T p.(His435Tyr)	c.368-1G>A	c.1303C>T p.(His435Tyr)	c.982G>A p.(Glu328Lys)
	Mutation 2	c.1543C>T p.(Arg515Cys)	c.1303C>T p.(His435Tyr)	c.1303C>T p.(His435Tyr)	c.1303C>T p.(His435Tyr)	c.1303C>T p.(His435Tyr)	c.368-1G>A	c.1303C>T p.(His435Tyr)	c.1303C>T p.(His435Tyr)
**Age at genetic diagnosis**		9	NA	59	6	8	NA	NA	3
**Origin**		(Basque Country) Spain (NE)	Morocco	(Madrid) Spain	(Madrid) Spain (	(Murcia) Spain (SE)	Morocco	Morocco	(Cádiz) Spain (S)
**Phenotype**		CIE	NA	LI	SHCB	CIE	LI	NA	CIE
**Collodion**		P	P	P	P	P	N	P	P
**Prematurity**		N	NA	N	P	N	N	N	P
**Ectropion**		N	NA	N	P	N	N	P	N
**Alopecia**		N	NA	N	N	P	N	N	N
**PPK**		P	NA	P	P	P	N	N	N
**Scales**	Size	B	S	B	S	S	V	NA	S
	Color	W	D	W	W	W	D	NA	W
**Altered Sweating**		N	P	P	N	N	P	NA	N
**Erythema**		P	N	N	P	P	N	NA	P
**PH**		P	P	P	P	P	P	NA	P
**Affected area**	Flexor	N	N	P	P	P	N	NA	P
	Extensor	N	P	P	N	P	P	NA	P
	Facial	P	N	P	P	N	P	NA	P
	Palmoplantar	P	P	P	P	P	N	NA	P
**TRT**		**TR**	**TR**	**TR &OR**	**TR**	**TR**	**TR**	**TR**	**TR**

Lamellar Ichthyosis (LI) Congenital Ichthyosiform Erythroderma (CIE) and SCHB (Self-Healing Collodion Baby).

^a^See pedigrees available in the supplementary data (S1-S4), numbers indicate the family while the combination of Roman and Arabic numerals denote the position of the patient within the pedigree. NE: North-East, C: Central, SE: South-East, S: South, P: positive, N: negative, NA. Not Available, B: Big, S: Small, W: Whitish, D: Dark, PPK: Palmoplantar keratoderma, PH: Palmar hyperlinearity, TRT: Treatment, TR: Topical Retinoids, **OR**: Oral Retinoids.

**Fig 2:**
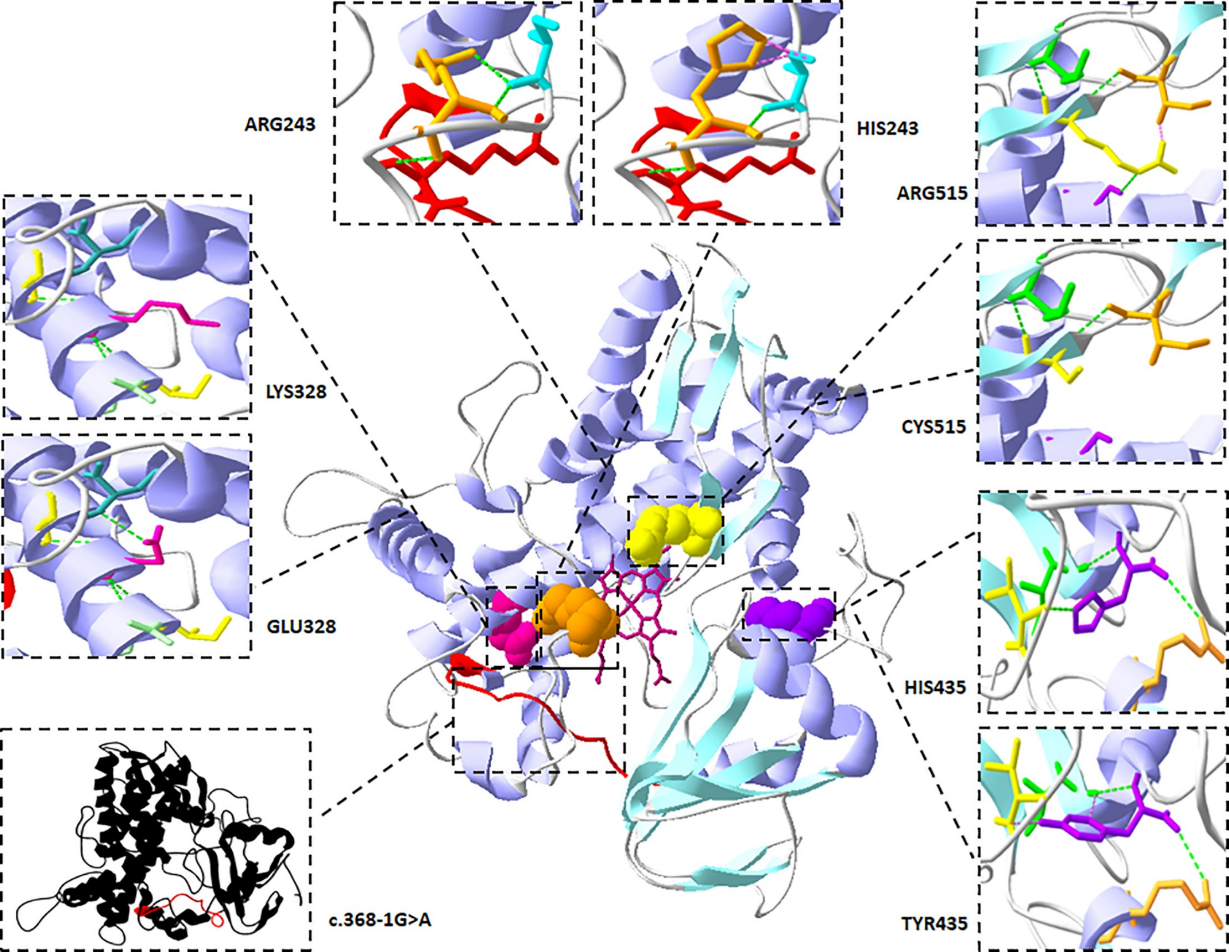
Ribbon diagram of the CYP4F22 protein with the position of the five pathogenic variants shaded in different colors. The heme prosthetic group is shown in the center of the figure, illustrated by a brown grid. Each variant has an inset to show the local environment of the wild type/altered residue. Green dots represent a strong H-bond while purple dots represent a clash (short distance repulsive energy).

The effect of the altered amino acids on the overall protein structure seems important for studying the effect of the mutations on the protein’s 3D structure and function (Fig 2). The potential impact of each mutation was predicted as follows:

- c.728G>A; p.(Arg243His), a bond between the arginine and a nearby serine located within the same loop is lost (S2 and S3 Figs). This could possibly alter the loop structure. Arg243 is conserved in all species examined from human to sheep (S4 Fig) and is predicted to be deleterious and disease causing by Mutation Taster and Sift predictors (See Table 2). Furthermore, the mRNA structure of mutant CYP4F22 predicted by mfold, revealed a different folding pattern compared to the wild type with a decrease in free energy (*dG = -2*.*68)*, increasing the RNA stability, which may affect the expression level of the protein (S5 Fig).

**Table 2:** Characteristics of the CYP4F22 mutations detected in our families.

Nucleotide Change	Aminoacidic change	Exon/Intron	Resultant Change	Mutation Type	In Silico Prediction					MAF	Reference
					*Mutation Taster*	*SIFT*	*Align GVGD*	*CADD Score*	*Splicing*		
**c.368-1G>A**		Intron 4	Skip of exon 5 very likely	Splice-Site				27.2	Predicted change at acceptor site 1 bps downstream:-100.0%	-	This study
**c.728G>A**	p.(Arg243His)	Exon 8	Highly conserved residue altered	Missense	Disease causing	Deleterious	Least likely to interfere with function	25.0		A = 4.6E-06	9
**c.982G>A**	p.(Glu328Lys)	Exon 9	Highly conserved residue altered	Missense	Disease causing	Deleterious	Least likely to interfere with function	26.6		A = 8.7E-07	33
**c.1303C>T**	p.(His435Tyr)	Exon 12	Highly conserved residue altered	Missense	Disease causing	Deleterious	Most likely to interfere with function	26.5		T = 7.7E-06	13
**c.1543C>T**	p.(Arg515Cys)	Exon 14	Moderately conserved residue altered	Missense	Disease causing	Deleterious	Most likely to interfere with function	29.7		-	This study

Mutation nomenclature: the Human Genome Sequence Variation guideline was followed. Reference sequences *CYP4F22* (NM_173483.3, NP_775754) were used for naming the nucleotide and protein variations respectively. A CADD score of 20 and above means that a variant is amongst the top 1% of deleterious variants in the human genome, a score of 30 means that the variant is in the top 0.1%. Percentages are the consensus values from 0 to 100 for Splicing prediction algorithms (HSF, MaxEnt and NNSPLICE). Available Minor Allele Frequencies (MAF) of European Non-Finnish population were taken from the gnomAD database (http://gnomad.broadinstitute.org/).

- c.982G>A; p.(Glu328Lys), the Glu328 residue is located in the middle of a helix and the side-chain forms three hydrogen bonds, one of which is with the side-chain of Leu144, in contrast, the Lys328 is unable to form this bond (S2 and S3 Figs). This residue is highly conserved (S4 Fig), suggesting it plays an important role in the stability of the protein. The variant does not alter the CYP4F22 mRNA structure (S6 Fig). Additionally, this substitution is predicted to be deleterious and disease causing by Mutation Taster and Sift predictors (See Table 2).

- c.1303C>T; p.(His435Tyr), the His435 residue is positioned in a loop and it has connections with other close residues (Ile 431, Val 466, Arg397) located in or near a beta sheet, loop and helix, respectively (S2 and S3 Figs). When histidine is replaced by tyrosine some of these connections are missing, altering the residue environment and the secondary structure of the protein. His435 is also conserved across all examined species (S4 Fig) and is very likely to be pathogenic according to all predictors (Table 2). The predicted mRNA structure of CYP4F22 is not disturbed by this mutation (S7 Fig).

- c.1543C>T; p.(Arg515Cys), arginine is a large amino acid that can interact with close residues establishing bonds with helix or loops, in contrast, cysteine is a small amino acid (S2 and S3 Figs). The replacement of arginine for cysteine would disrupt some of these relations, in turn disturbing the secondary structure of the protein. Arg515 is conserved in all species from human to sheep (S4 Fig) and all algorithms *predicted* the *pathogenicity* of *this variant (Table 2).* The variation was predicted not to affect the structure of mRNA (S8 Fig).

- c.368-1G>A is predicted to alter the splicing process by skipping of the fifth exon of the *CYP4F22* gene (Table 2). Thus the protein structure would be altered by the loss of at least one loop (the missing loop is colored red in Fig 2).

### Clinical and genetic characteristics of patients with *CYP4F22* mutations

Two of the eight patients with *CYP4F22* mutations presented a clinical phenotype of LI, three of the eight affected individuals had the CIE phenotype, and only one patient was diagnosed with SCHB. Clinical diagnosis was not available for two patients (Patients 34 and 127). The most common symptoms were collodion membrane at birth, palmar hyperlinearity and whitish scales all over the body (see Table 1). The majority of patients were taking topical retinoids. Contrastingly, the presence of alopecia, ectropion and premature birth were infrequent among these patients. Whitish small scales were observed more often than big and dark scales (Table 1).

Among patients with LI, we found two different types of mutations: one individual was homozygous for splice-site mutation c.368-1G>A, while the other one was a compound heterozygous carrier of two different missense mutations [c.1303C>T; p.(Arg435Tyr) and c.728G>A; p.(Arg243His)]. All patients with CIE had missense mutations. Moreover, the homozygous c.1303C>T mutation was present in patients with either SCHB (family 67) or CIE phenotypes (family 94), as shown in Table 1.

### Haplotype construction and TMRCA estimation

We constructed the haplotypes flanking the *CYP4F22* locus in eleven c.1303C>T carrier chromosomes. A prevalent haplotype, 3-2-5-8, over a 2.52 Mb interval that stretches from marker D19S840 to D19S917 was identified among Spanish *CYP4F22* chromosomes with the c.1303C>T mutation (Fig 1). This haplotype was significantly more frequent in patients (three out of seven haplotypes) than in controls (one haplotype in two hundred) (*p* = 0.0000869). The 4-6-5-5-8 haplotype ranging from markers D19S221 to D19S917 was shared between the two patients with Moroccan origin (families 34 and 127), both of whom were homozygous for the c.1303C>T mutation. A common core haplotype of 1.2Mb (5–8) was present in almost all carrier patients (five patients, eight chromosomes; Fig 1). We determined the TMRCA of carrier chromosomes with Spanish origin, by applying both single marker and haplotype sharing methods (Table 3).

**Table 3:** TMRCA and mutation age estimations.

TMRCA		MUTATION AGE (generations)				
**Algorithms**	Estimated time		DMLE		BDMC2.1	
**Bergman**	26 (13–40)		Growth Rate r = 0.0748	Growth Rate r = 0.05	Growth Rate r = 0.0748	Growth Rate r = 0.05
**Risch**	39 (24–54)	Values of (f)	Estimated generations	Estimated generations	Estimated generations	Estimated generations
**Yan**	23 (10–37)	*f* = 0.000299	112.33 (88.68–157.00)	162.63 (125.07–229.70)	130 (129.92–130.08)	190 (189.97–190.03)
**Genin**	23 (13–44)	*f* = 0.000203	119.79 (93.19–164.12)	171.02 (129.45–234.05)	130 (129.93–130.07)	200 (199.96–200.04)
**Gandolfo**	34 (14–90)	*f* = 0.000232	117.46 (91.76–161.37)	164.51(128.53–226.66)	130 (129.92–130.08)	200 (199.99–200.01)
***All estimators***	*29 (*23–35*)*	Mean estimations	116.53 (120.85–112.21)	166.05 (161.07–171.04)	130 (129.92–130.08)	196.67 (190.13–203.20)

Results are given in number of generations, with a confidence interval of 95% for the mean. The mutation age was calculated using a variable proportion of mutated chromosomes (*f*) and a variable population growth rate (*r*). See Materials and Methods section for a detailed description.

Using haplotype sharing methods, the TMRCA was estimated to be 23 generations (95% CI 13–44) using Genin´s approximation, while Gandolfo´s algorithm showed a mean of 34 generations (95% CI 14–90). We could also see differences in the estimates from the three single marker methods, depending on the algorithm applied: 26 generations (95% CI 13–40) for Bergman´s estimator, 39 generations (95% CI 24–54) according to Risch´s estimator and 23 generations (95% CI 10–37) with Yan´s equation. By averaging the estimates of all methods, the TMRCA of c.1303C>T mutation carrier patients with Spanish ancestry would date back 29 (95% CI 23–35) generations. Assuming an average generation time of 25 years, this would mean that carrier families derived from a common ancestor who lived approximately 725 years ago (Table 3).

### Dating the c.1303C>T mutation

We estimated the age of the c.1303C>T mutation using two mathematical approaches, DMLE+ and BDMC21. According to DMLE+ (using an average population growth of *r* = 0.05 and different values of sampled population (*f* = 2.99x10^-4^, *f* = 2.03x10^-4^, *f* = 2.32x10^-4^), the mean posterior age estimate was 162.63 (95% CI 125.07–229.70), 171.02 (95% CI 129.45–234.05) and 164.51 (95% CI 128.53–226.66) generations, respectively. This approximately equates to 4075 to 4275 years ago, assuming a generation time of 25 years. Lower estimates of 112.33 (95% CI 88.68–157.00), 119.79 (95% CI 93.19–164.12) and 117.46 (95% CI 91.76–161.37) were obtained for the same *f* values by using a growth rate of *r* = 0.0748. These figures would correspond to a mutation age range of 2800 to 3000 years.

When using the BDMC2.1 approach, the number of generations increases. The analyzed range of *f* gives an estimation of 130 (95% CI 129.92–130.08) for a growth rate of *r* = 0.0748, thus the mutation would date to 3250 years. A growth rate of *r* = 0.05 with the same *f* values gives an estimated age of 196.67 (95% CI 190.13–203.20). This would indicate that the c.1303C>T mutation could have arisen approximately 4925 years ago. Data shown in Table 3.

## Discussion

In the present study, we report five different *CYP4F22* mutations found in eight ARCI families with two origins (Spanish and Moroccan). Four mutations were missense and one was splice-site, which is in line with the mutation type frequencies already present in the literature which include: twenty-six missense (60%), seven splice-site (14%), five nonsense (10%), six frameshift (12%), one indel (2%) and one gross deletion (2%), implying that ARCI patients with mutations in *CYP4F22* are more likely to carry missense mutations than any other type of pathogenic variants [13,16,33–44]. It is interesting to note that, despite affecting well-conserved residues across multiple species, our 4 coding mutations are not located within the putative functional domains of the protein [39,45]. Furthermore, we found that only five mutations [13,35,40,44], from the forty-six reported to date, were within these domains, which suggests that other regions of the protein may have critical functions.

Clinical presentation varied significantly among these patients, but the most common symptoms were: collodion membrane at birth, fine whitish scales and affected palmoplantar area [16,36,38,44,45]. No genotype-phenotype correlation between the location or type of mutation, and major clinical features, was evident in our patients. This could indicate that other factors, either external, such as environment or lifestyle, or internal, such as the genetic background of each individual, could modulate the penetrance of these mutations.

To date, *CYP4F22* mutations have been reported in different populations: Italian, Algerian, Scandinavian, Iranian, Spanish, etc [13,15,16,36,43]. The mutation frequency varies among different populations, in Scandinavian countries, Iran and Italy the percentages vary from 2% to 6% of all ARCI-related mutations [16,36,43], whereas in populations where a founder effect seems likely, the frequency reaches 10–12% [35,44]. Thus, Bučková *et al* proposed a founder effect for the c.59dupG mutation in the Czech population, while Hotz *et al* suggest a similar effect in the case of c.1303C>T in the Algerian population [35,44]. In our cohort, *CYP4F22* mutations represent 9% (8 out of 92) of the total ARCI cases. This percentage is similar to those reported by Bučková *et al* and Hotz *et al* and this could be due to a founder effect for c.1303C>T in the Spanish population. The c.1303C>T mutation has been previously reported in a total of seventeen families, including one Italian case [43], thirteen families from Algeria [13,35], two patients from Germany and Cape Verde [35], two Persian, one Turkish family [36] and a Spanish individual with SHCB [15]. This last patient was also included in the current study. The majority of the families reported to carry this mutation come from the same area of Algeria [35].

The high frequency of the variant c.1303C>T detected in our patients, led us to investigate if the observed alleles share a common origin. The data obtained from the study of ten STR markers revealed the existence of a 2.52Mb major haplotype among carrier patients with Spanish ancestry confirming the hypothesis of a recent common ancestor. A smaller core haplotype of 1.2Mb was shared by almost all c.1303C>T carrier chromosomes which suggests that Spanish and Moroccan families could be descendants from a further common ancestor.

To further address this issue, we estimated the TMRCA of Spanish carrier families with two different approaches, and obtained a median of 29 generations. This estimation would date their common ancestor to the XIII century, thus, it is possible that this ancestor could have African origin, considering the prevalent Berber origin (including here Morocco and Algeria) of the Arab groups invading southern Europe, during the VIII-XV century AD [46].

In order to study the history of the *CYP4F22* c.1303C>T substitution, we estimated the mutation age, which is not *necessarily the same* as the *TMRCA*, by using two programs: BDMC21 and DMLE+. These methods are known to be highly dependent on demographic parameters. In fact, when two different growth rates are used, the estimated allelic age varies considerably, while different proportions of mutation-bearing chromosomes does not seem to lead to much potential variability, especially in the case of BDMC21. Regarding the age of mutation estimation, we consider that results derived from DMLE calculations are more accurate. This is due to the sophistication of the algorithm, which accounts for more variables and complex data compared with BDMC21. We also believe that a growth rate of 0.05 is more rigorous than growth rate of 0.0748, since this founder mutation is also present in other European countries and is not only confined to Spain.

Despite the possible limitations, it is plausible to hypothesize that the *CYP4F22* c.1303C>T variant originated approximately 2925 to 4925 years ago in a Neolithic population from North Africa and later, in the XIII century, it was introduced to the Iberian Peninsula during the Islamic invasions in Southern Europe and specifically in the Spanish territory.

In summary, the present study reports five *CYP4F22* mutations derived from the genetic analysis of eight ARCI families. Two of the identified mutations were novel, c.368-1G>A and c.1543C>T; p.(Arg515Cys), increasing the number of *CYP4F22* mutations currently listed. We used *in silico* approaches to predict their possible effect on the protein function. Although there was some variation, we found that the presence of collodion membrane at birth, palmar hyperlinearity and palmoplantar affected area were the most common symptoms among our patients with *CYP4F22* mutations. c.1303C>T; p.(His435Tyr) was the most prevalent mutation, identified in our families and was confirmed to be a consequence of a founder effect in the Spanish population. These families originated from a single ancestor with probable African ancestry who lived 29 generations ago and the mutation first appeared in the Neolithic era.

## Supporting information

S1 Table Primers used for sequencing the ten polymorphic microsatellite markers. (DOCX)

S2 Table Linkage disequilibrium analysis. Values for the TMRCA calculations according to Bergman, Risch and Yan algorithms for each marker with the correction proposed by Labuda et al. TMRCA age estimation was also calculated using the algorithms developed by Gandolfo et al and Genin et al. Labuda correction is also expressed in generations. θ: recombination fraction according to Haldane mapping function, PD: frequency of the founder allele in the normal population, PN: frequency of the founder allele in the disease population.
(DOCX)

S1 Fig Pedigree of families 31, 57, 67 and 94. (TIF)

S2 Fig Local environment and interactions of the wild type residues. Arg243(Orange), Arg328(Magenta), His435(Violet) and Arg515(Yellow). Green dots represent a strong H-bond while purple dots represent a clash (short distance repulsive energy).
(TIF)

S3 Fig Local environment and interactions of the mutated residues. His243(Orange), Lys328(Magenta), Tyr435(Violet) and Cys515(Yellow). Green dots represent a strong H-bond while purple dots represent a clash (short distance repulsive energy).
(TIF)

S4 Fig Comparison of partial amino acid sequence of human CYP4F22 across different species. Red shaded amino acids indicate the conserved residue affected by the four missense mutations.
(TIF)

S5 Fig The secondary mRNA structure of wild type (c.728G) and mutant (c.728A) CYP4F22. Predicted by mfold online software (simulated under standard parameters). The altered nucleotide is shaded in yellow.
(TIF)

S6 Fig The secondary mRNA structure of wild type (c.982G) and mutant (c.982A) CYP4F22. Predicted by mfold online software (simulated under standard parameters).
(TIF)

S7 Fig The secondary mRNA structure of wild type (c.1303C) and mutant (c.1303T) CYP4F22. Predicted by mfold online software (simulated under standard parameters).
(TIF)

S8 Fig The secondary mRNA structure of wild type (c.1543C) and mutant (c.1543T) CYP4F22. Predicted by mfold online software (simulated under standard parameters).
(TIF)
