# Supplementary material for: Novel CYP4F22 mutations associated with autosomal recessive congenital ichthyosis (ARCI). Study of the CYP4F22 c.1303C>T founder mutation
Source: PLoS One. 2020 Feb 18;15(2):e0229025. doi: 10.1371/journal.pone.0229025 (PMC7028276; doi:10.1371/journal.pone.0229025)
Supplement: S1 Table — (DOCX) [file pone.0229025.s001.docx]

**Table S1**. Primers used for sequencing the ten polymorphic microsatellite markers

| **Primers** | **Oligonucleotide sequences** | **Product lenght (bp)** |
| --- | --- | --- |
| **D19S568F** | GCAGCACTGAATATAGTACCAC | 193 |
| D19S568R | AATTGTCTTGGCTAGAGATTCT |  |
| **D19S415F** | TACTGTTACTGTTGCCTTGAGT | 240 |
| D19S415R | GTACCCACTCCTTTCTAGTAGTATG |  |
| **D19S221F** | AGCAAGACTCTGACTCAACAAA | 291 |
| D19S221R | GCCAAAGGTTTTCTGATATACA |  |
| **D19S212F** | AGTGTGACTGTCCTTGTGCTAA | 357 |
| D19S212R | AGCAAAAATACAAAAATTAGCC |  |
| **D19S929F** | CCCTTCTCCAAGATCAGTACGTGGG | 400 |
| D19S929R | ATCTCTGTCTCCATCATGGCCAACA |  |
| **D19S840F** | GATAGGCCAAGACTGTCTAAAAC | 156 |
| D19S840R | GAATCAAGACTTGCTGTATGACA |  |
| **D19S407F** | AAATTAGCCGGGCGTGATGGTGC | 217 |
| D19S407R | GCCTGCAACAGAGGGTTGTTTGTT |  |
| **D19S593F** | GAGATGACAGATGAAGAGATGG | 286 |
| D19S593R | GATGCTACTCAATACCCTACAG |  |
| **D19S917F** | GACATAACACAGGTGCAGATAC | 327 |
| D19S917R | GAATAGACTCCCTGGATCATAT |  |
| **D19S581F** | TACAAAAAATTTCAAGAATTAGCT | 370 |
| D19S581R | GAAGTGTTTACTATTATGGGTGTC |  |
