# Supplementary material for: Novel CYP4F22 mutations associated with autosomal recessive congenital ichthyosis (ARCI). Study of the CYP4F22 c.1303C>T founder mutation
Source: PLoS One. 2020 Feb 18;15(2):e0229025. doi: 10.1371/journal.pone.0229025 (PMC7028276; doi:10.1371/journal.pone.0229025)
Supplement: S2 Table — Values for the TMRCA calculations according to Bergman, Risch and Yan algorithms for each marker with the correction proposed by Labuda et al. TMRCA age estimation was also calculated using the algorithms developed by Gandolfo et al and Genin et al. Labuda correction is also expressed in generations. θ: recombination fraction according to Haldane mapping function, PD: frequency of the founder allele in the normal population, PN: frequency of the founder allele in the disease population. (DOCX) [file pone.0229025.s002.docx]

**Table S2**. Linkage disequilibrium analysis

Values for the TMRCA calculations according to Bergman, Risch and Yan algorithms for each marker with the correction proposed by Labuda et al. TMRCA age estimation was also calculated using the algorithms developed by Gandolfo et al and Genin et al. Labuda correction is also expressed in generations. θ: recombination fraction according to Haldane mapping function, PD: frequency of the founder allele in the normal population, PN: frequency of the founder allele in the disease population.

| **Markers** | **Haldane (θ)** | **Founder allele** | **PD** | **PN** | **Labuda correction** | **TMRCA in generations** | | |
| --- | --- | --- | --- | --- | --- | --- | --- | --- |
|  |  |  |  |  |  | **Bergman** | **Risch** | **Yan** |
| D19S581 | 0.09 | 3 | 0.43 | 0.20 | -3 | 13 | 70 | 10 |
| D19S221 | 0.06 | 8 | 0.57 | 0.25 | 3 | 15 | 26 | 12 |
| D19S840 | 0.04 | 3 | 0.86 | 0.25 | 10 | 6 | 9 | 6 |
| D19S415 | 0.01 | 2 | 1 | 0.44 | 23 | - | - | - |
| D19S917 | 0.01 | 5 | 0.86 | 0.38 | 27 | 26 | 26 | 25 |
| D19S929 | 0.01 | 8 | 0.71 | 0.41 | 22 | 47 | 47 | 40 |
| D19S593 | 0.03 | 1 | 0.71 | 0.34 | 11 | 17 | 50 | 15 |
| D19S212 | 0.05 | 7 | 0.57 | 0.26 | 5 | 17 | 15 | 14 |
| D19S407 | 0.08 | 2 | 0.43 | 0.09 | -2 | 12 | 24 | 10 |
| D19S568 | 0.12 | 5 | 0.43 | 0.37 | -7 | 19 | 17 | 10 |
