# Supplementary figures and images for: Novel CYP4F22 mutations associated with autosomal recessive congenital ichthyosis (ARCI). Study of the CYP4F22 c.1303C>T founder mutation
Source: PLoS One. 2020 Feb 18;15(2):e0229025. doi: 10.1371/journal.pone.0229025 (PMC7028276; doi:10.1371/journal.pone.0229025)

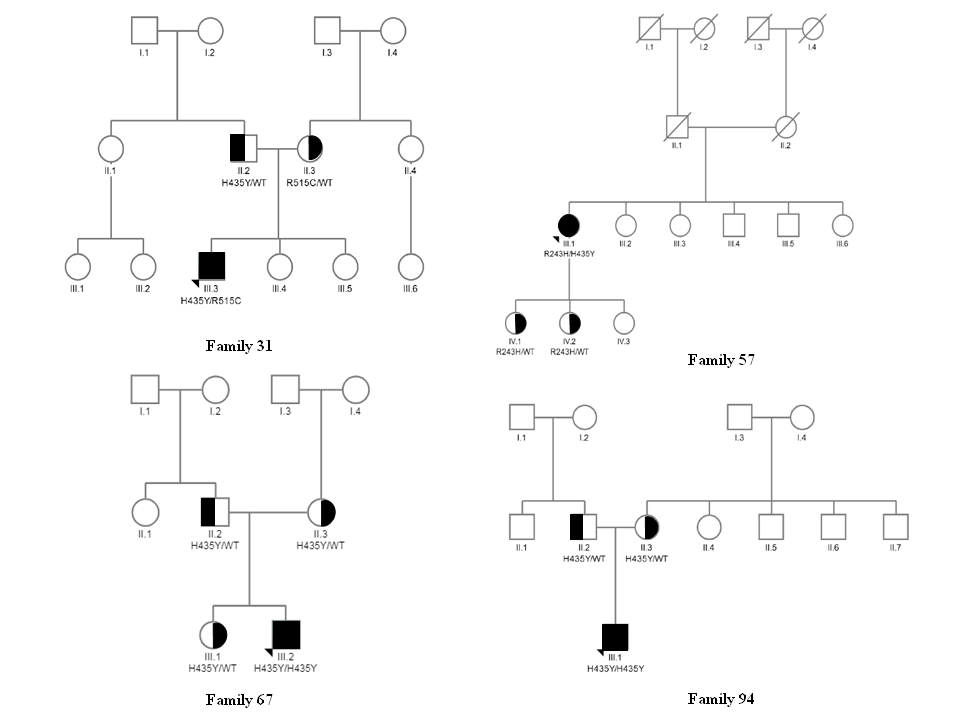

Supplement: S1 Fig — (TIF) [file pone.0229025.s003.tif]

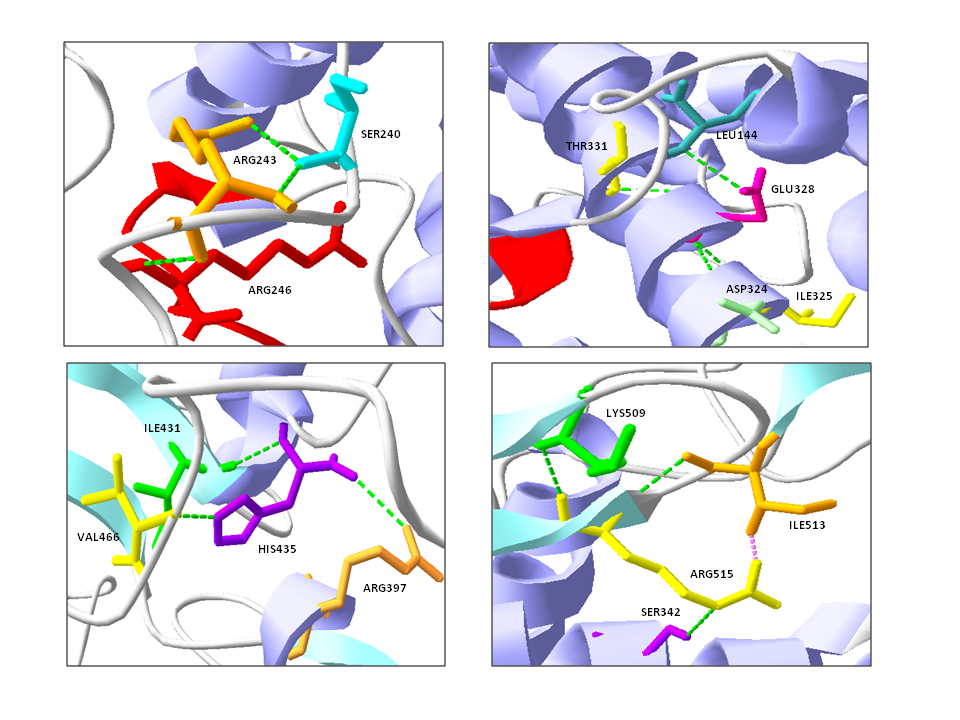

Supplement: S2 Fig — Arg243(Orange), Arg328(Magenta), His435(Violet) and Arg515(Yellow). Green dots represent a strong H-bond while purple dots represent a clash (short distance repulsive energy). (TIF) [file pone.0229025.s004.tif]

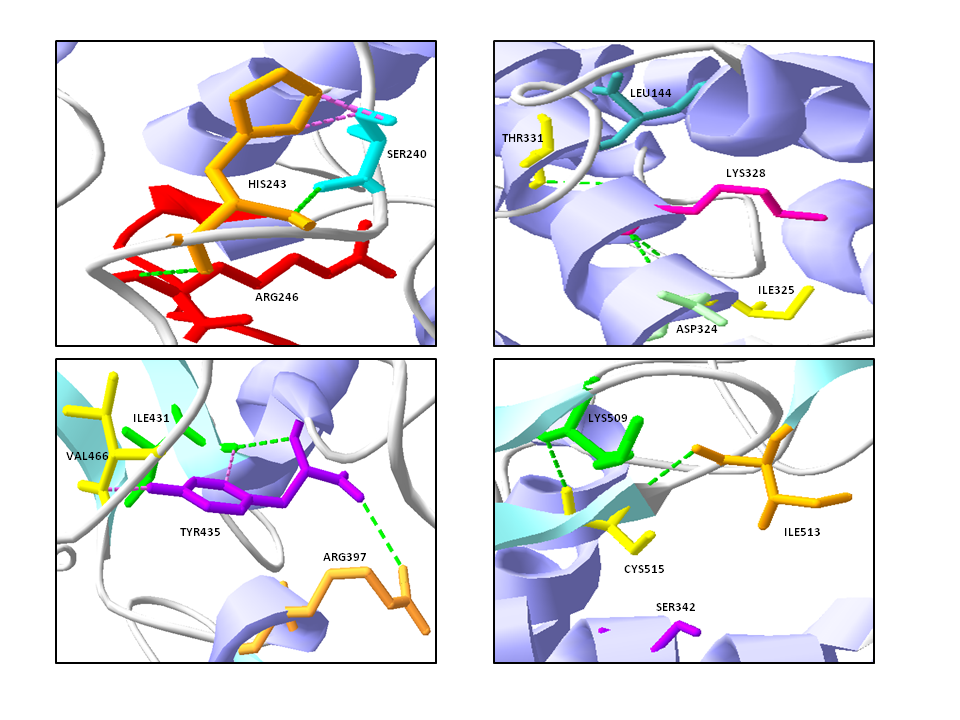

Supplement: S3 Fig — His243(Orange), Lys328(Magenta), Tyr435(Violet) and Cys515(Yellow). Green dots represent a strong H-bond while purple dots represent a clash (short distance repulsive energy). (TIF) [file pone.0229025.s005.tif]

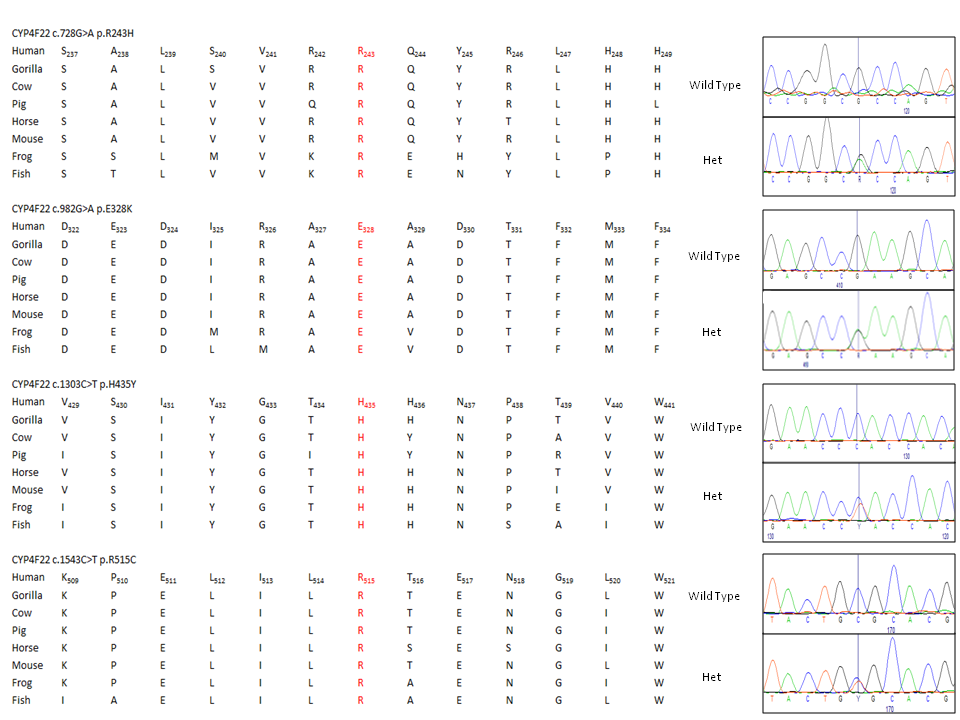

Supplement: S4 Fig — Red shaded amino acids indicate the conserved residue affected by the four missense mutations. (TIF) [file pone.0229025.s006.tif]

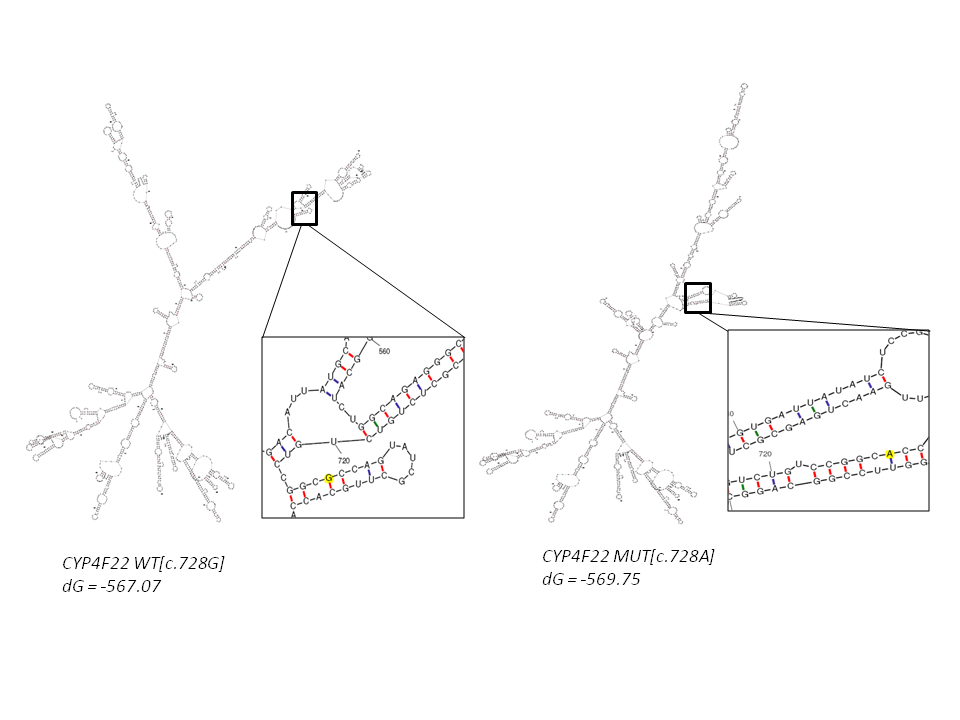

Supplement: S5 Fig — Predicted by mfold online software (simulated under standard parameters). The altered nucleotide is shaded in yellow. (TIF) [file pone.0229025.s007.tif]

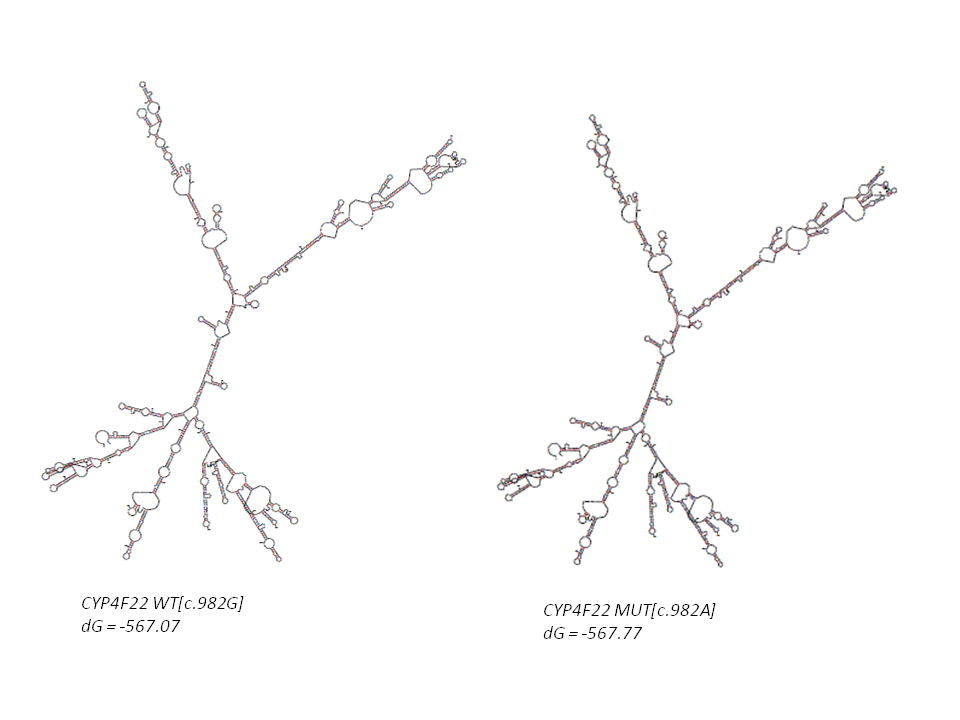

Supplement: S6 Fig — Predicted by mfold online software (simulated under standard parameters). (TIF) [file pone.0229025.s008.tif]

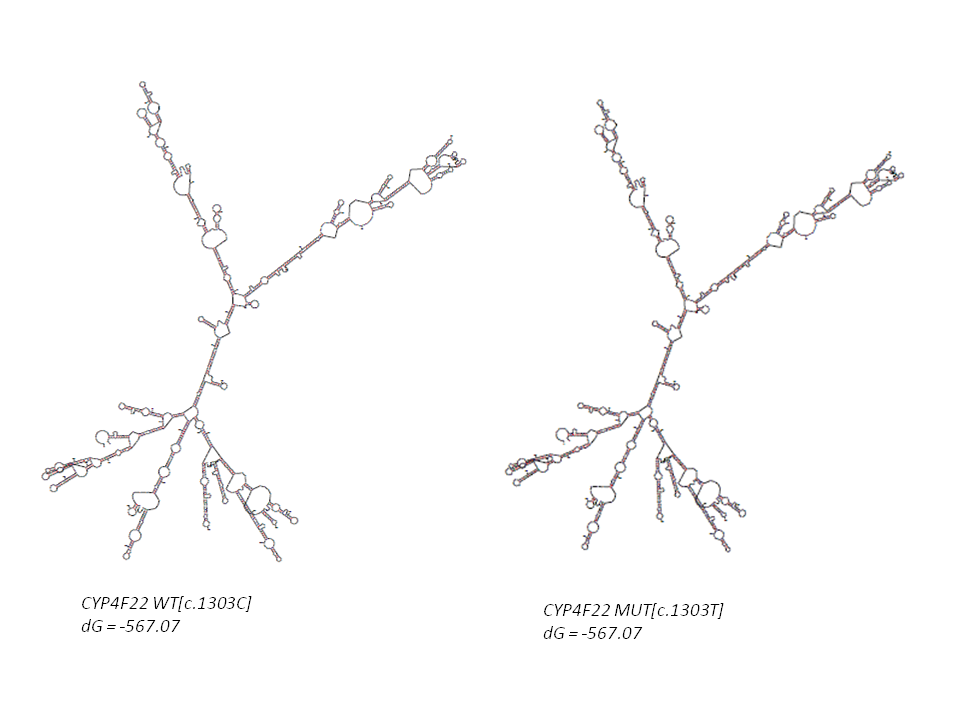

Supplement: S7 Fig — Predicted by mfold online software (simulated under standard parameters). (TIF) [file pone.0229025.s009.tif]

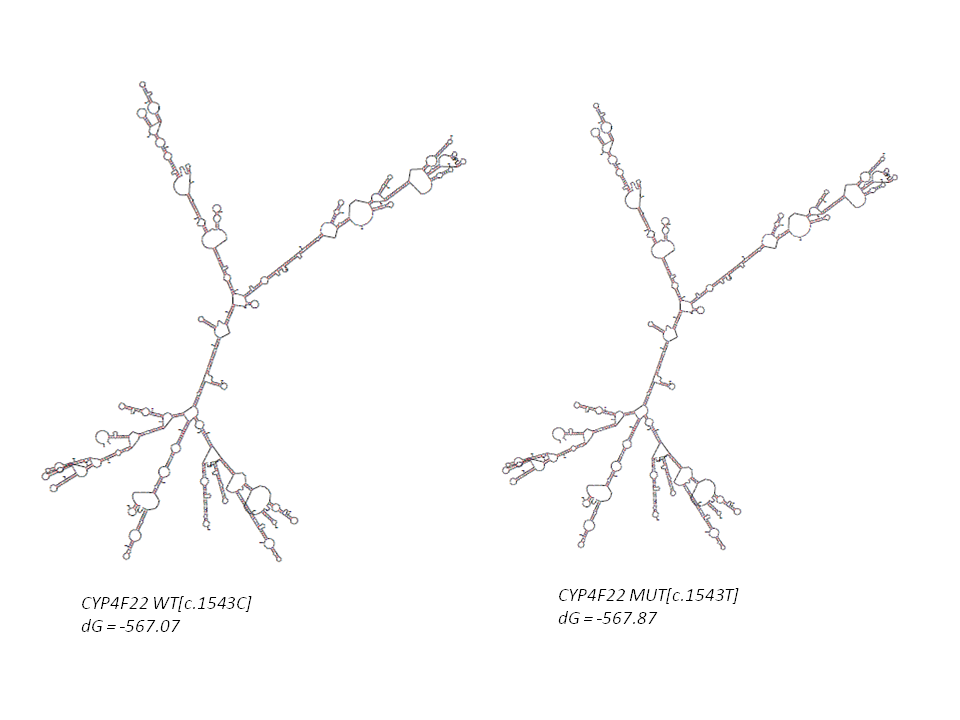

Supplement: S8 Fig — Predicted by mfold online software (simulated under standard parameters). (TIF) [file pone.0229025.s010.tif]
